# Supplementary material for: Automated bone mineral density prediction and fracture risk assessment using plain radiographs via deep learning
Source: Nat Commun. 2021 Sep 16;12:5472. doi: 10.1038/s41467-021-25779-x (PMC8446034; doi:10.1038/s41467-021-25779-x)
Supplement: Supplementary file 1 — Supplementary Information [file 41467_2021_25779_MOESM1_ESM.pdf]

Supplementary methods

DXA measurement

The densitometer is operated by multiple radiology technicians. In CGMH, all technicians need to be certified to operate the densitometer. The scans were analyzed following recommendations issued by the Taiwan Radiological Society[46] (amended from International Society for Clinical Densitometry, ISCD).[47] The recommendation briefly: 1. All DXA technicians need to complete the ISCD training course (basic and advanced) and operate at least 120 cases of DXA examination before the independent operation of DXA. 2. All technicians need to obtain the SOP provided by the manufacturer and conduct BMD measurements accordingly. 3. All DXA scanners need to have a detailed SOP at the examination site, which needs to be updated regularly and reviewed by relevant professionals. 4. All DXA scanners need to comply with the local radiation safety guideline. 5. Spine phantom BMD measurement is performed regularly to document the stability of DXA performance over time. BMD values must be maintained within an error of ±1.5%. A monitoring plan is needed for a correction approach when the error has been exceeded. 6. All DXA technicians need to establish a personal least significant change (LSC). The current recommendation is that the personal LSC should be within 5.3% for the lumbar spine, 5.0% for the whole hip, and 6.9% for hip neck BMD measurement.

Deep Adaptive Graph (DAG)

In DAG, the anatomical landmarks are formulated as a graph, $\mathrm{G} = (\mathrm{V}, \mathrm{E}, \mathrm{F})$, where the vertices V represent the landmarks, the edges E represent the relationships between them, and the features F encode visual patterns in the neighborhoods of the vertices. For a specific input image, the graph vertices are first initialized in the image using the mean shape of the anatomy, and a neural network is used to

displace the vertices from the initial position to the target anatomy in the image. By formulating the anatomical landmarks as a graph and modeling their displacements by the convolutional neural network–graph convolutional network (GCN), DAG can effectively exploit the structural information and shape prior to the anatomical landmarks. Therefore, DAG provides robust and accurate anatomical landmark detection on both hip and spine radiographs.

The neural network consists of a convolutional neural network to encode the input image to produce graph features F, and a GCN to process the graph to locate its vertices. Specifically, the GCN consists of two parts: a global transformation GCN and multiple local refinement GCNs. The global transformation GCN produces an affine transformation matrix, M, which brings the initial graph vertices closer to the target. The transformed vertices are written as follows:

$$\text{Equation (4): } V^1 = \{{v_i}^1\} = \{M{v_i}^0\},$$

where $V^0 = \{{v_i}^0\}$ and $V^1 = \{{v_i}^1\}$ denote the initial graph vertices before and after the estimated affine transformation, respectively. The local refinement GCNs then iteratively estimate the displacements of the graph vertices V^1. In each iteration, the vertices are displaced as follows:

$$\text{Equation (5): } {v_i}^{t+1} = {v_i}^t + \Delta {v_i}^t,$$

where $\Delta {v_i}^t$ is the displacement estimated by the local refinement GCN at the t-th step.

The training loss is calculated for both the global transformation GCN and the local refinement GCNs. Because the goal of global transformation GCN is to locate the anatomy coarsely, the following margin loss is used:

$$\text{Equation (6): } L_{global} = \left[\frac{1}{N}\sum_{i\in N}|v_i{}^1 - v_i| - m\right]_+ ,$$

where $[u]_+ = \max(0, u)$; $v_i{}^1$ and v_i Denote the globally transformed and ground truth vertices, respectively; and m is a hyperparameter representing a margin that aims to achieve high robustness for coarse landmark detection and forgive small errors. To encourage the local refinement GCNs to learn a precise localization, the L1 loss is directly applied to all vertices after the refinements, written as follows:

$$\text{Equation (7): } L_{local} = \frac{1}{N}\sum_{i\in N}|v_i{}^T - v_i|,$$

where $v_i{}^T$ denotes the vertices after the last local refinement GCN. The graph edge weights are treated as learnable parameters, which are initialized randomly at the beginning of training and updated via back-propagation during training.

Implementation details

Deep learning models were developed on a workstation with a single Intel Xeon E5-2650 v4 CPU @ 2.2 GHz, 128 GB RAM, and 4 NVIDIA TITAN V GPUs running Ubuntu 18.04 LTS. All code used in this study was developed in Python v3.6, and deep learning models were implemented using PyTorch v1.3. Image preprocessing was performed using the Python Imaging Library. ImageNet pre-trained weights were used to initialize the backbone network VGG-16 block. The Adam optimizer was used to train the model for 200 epochs with a batch size of 8, a starting learning rate of 1e−4, and a weight decay of 1e−4. The learning rate was reduced to 1e−5 after the first 100 training epochs. The following augmentations are performed during training: 1) color jittering on both the brightness (+-0.2) and contrast (+-0.2). 2) random up-down and left-right flipping, 3) random affine transformation (rotation +-30, shear +- 0.2, translation +- 25 pixels, scaling

+-10%). The trained model was evaluated on the validation set after each training epoch, and the model with the highest validation correlation coefficient r-value was selected as the best model.

Supplementary Figure 1. Flowchart of the study population.

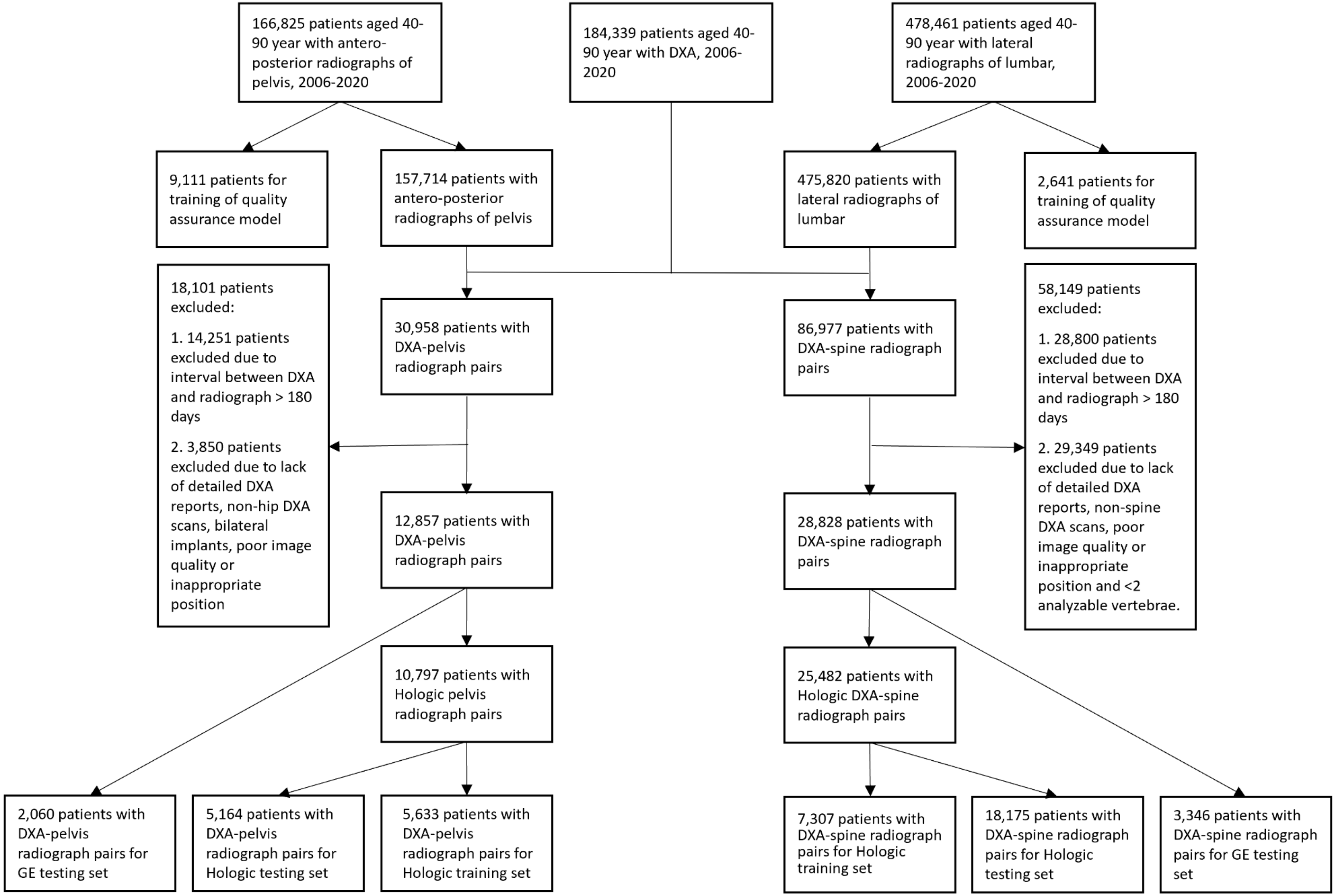

Supplementary Figure 2 Comparison of the receiver-operator curve between FRAX-AI (Navy blue line) and FRAX-NB tools (Maroon line) to classify high 10-year risk groups for (a) major fracture (>=20%) and (b) hip fracture (>=3%)

(a)

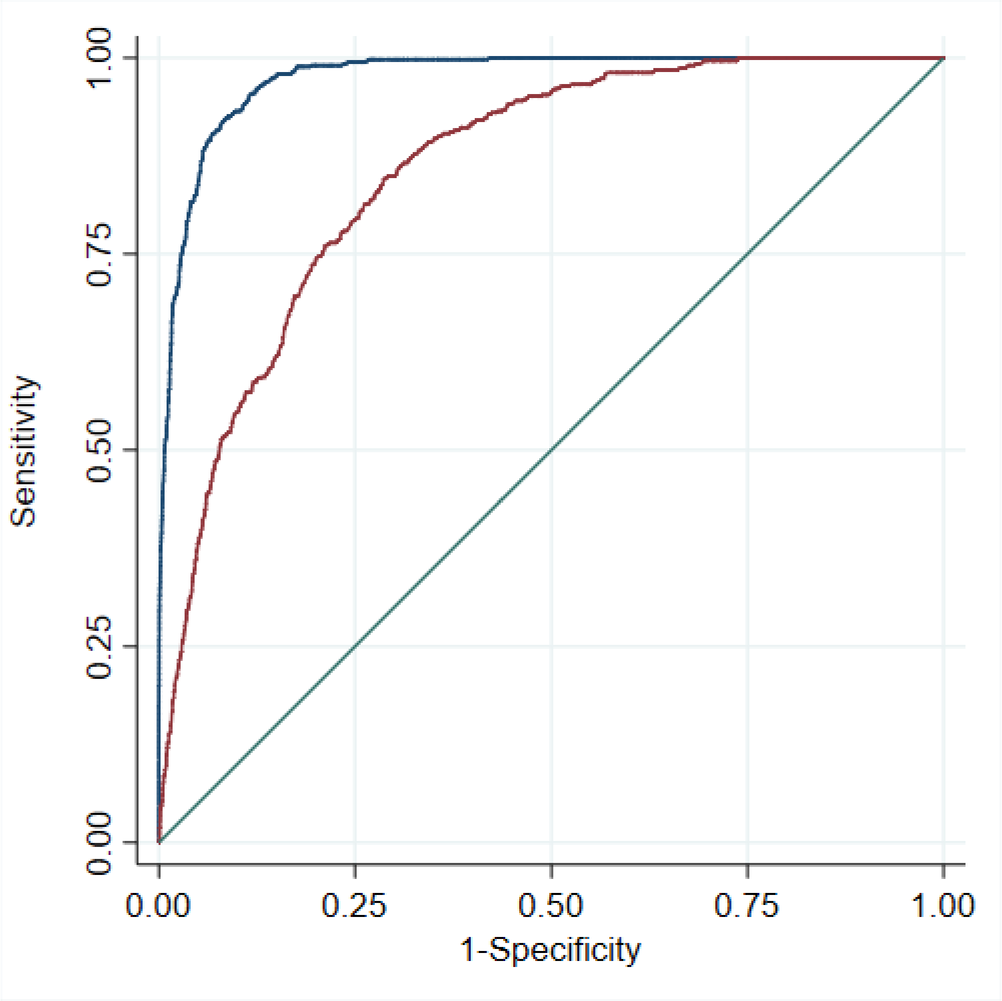

(b)

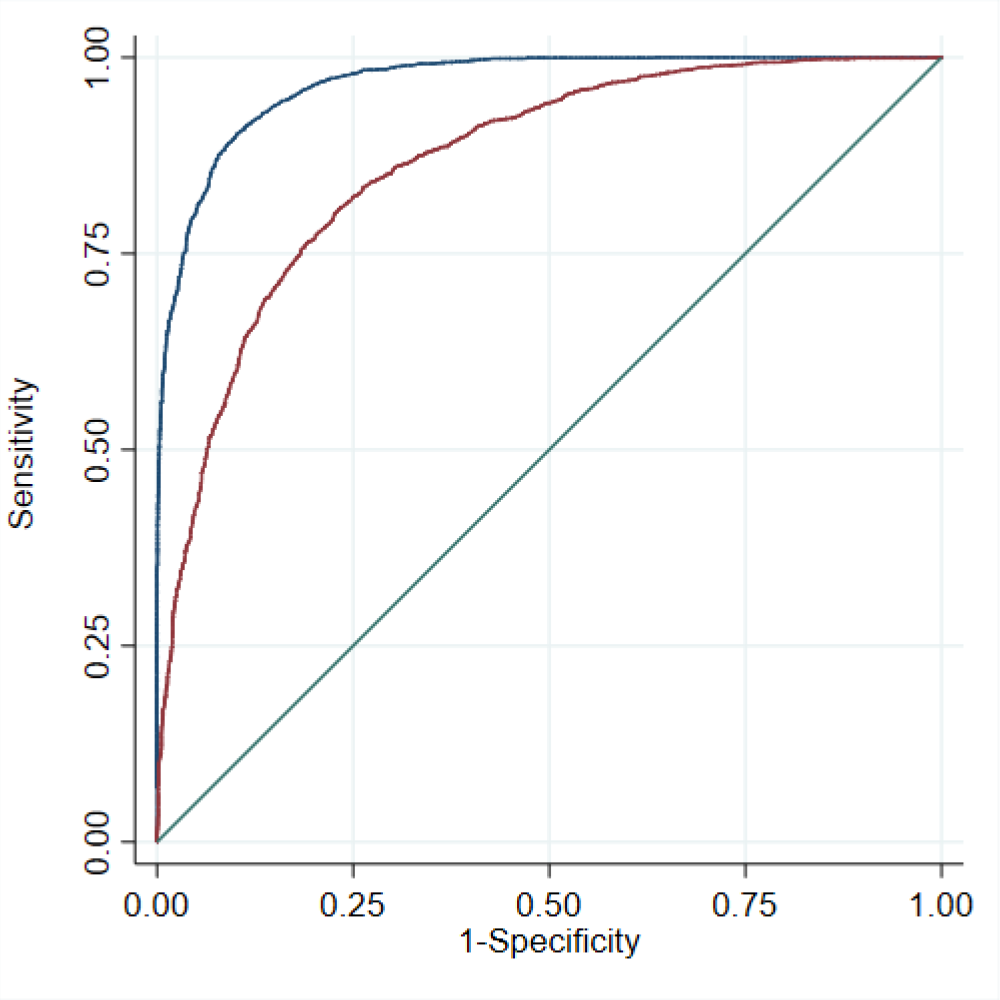
Sensitivity
0.00
0.25
0.50
0.75
1.00
0.00
0.25
0.50
0.75
1.00
1-Specificity

Supplementary Figure 3 Comparison of the precision-recall curve between FRAX-AI (Navy blue line) and FRAX-NB tools (Maroon line) to classify high 10-year risk groups for (a) major fracture (>=20%) and (b) hip fracture (>=3%)

(a)

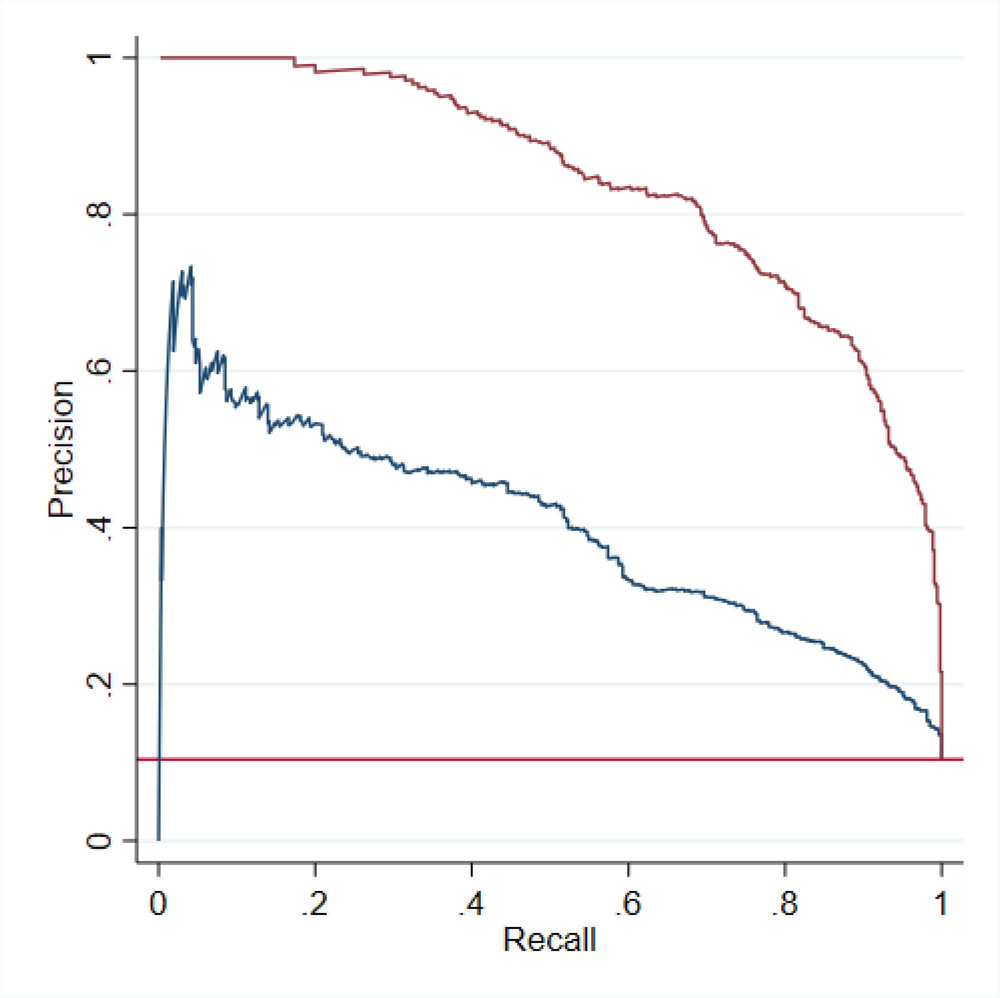

(b)

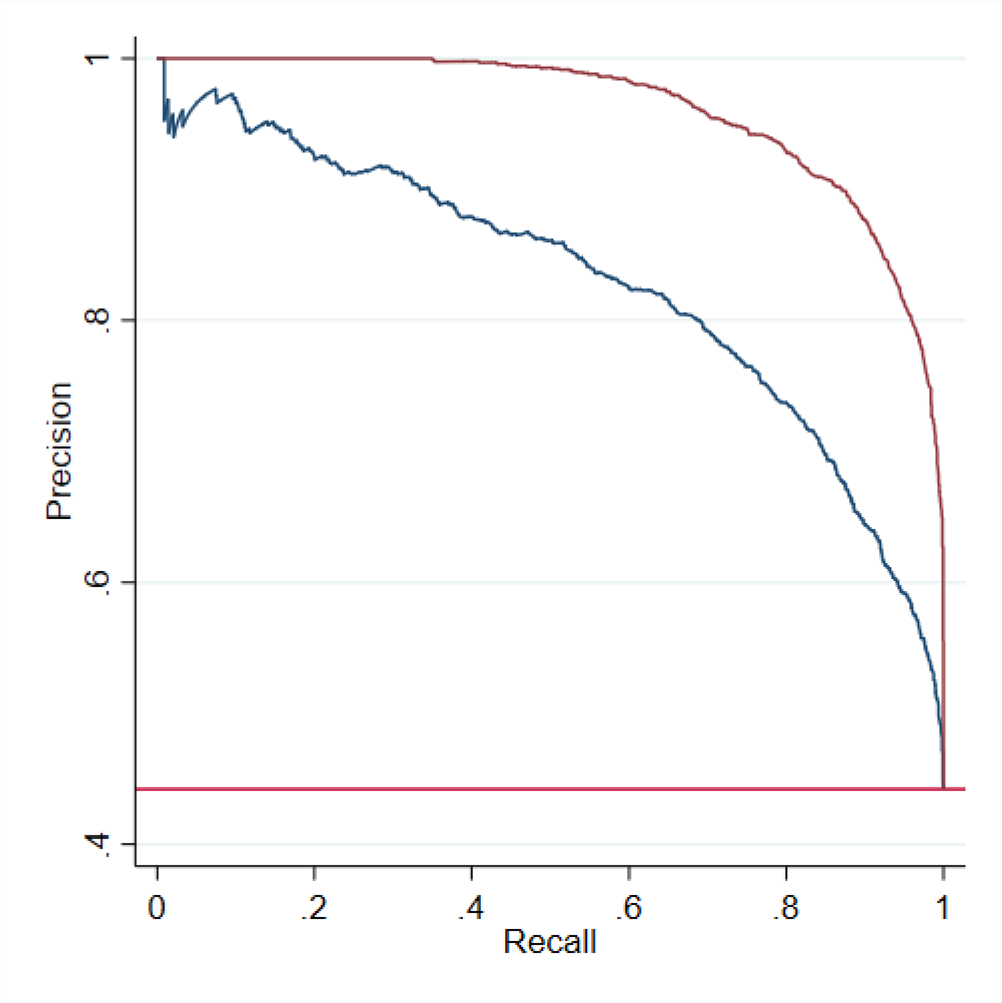
Precision
1
.8
.6
.4
0
.2
.4
.6
.8
1
Recall

Supplementary Figure 4 The calibration plots for predicted-measured BMD. (a) 2060 pairs of predicted-measured hip BMD (2060 patients) and (b) 11027 pairs of predicted-measured lumbar vertebral BMD (3346 patients). The DXA BMD was based on the measurement by the GE Lunar iDXA. Each point represents a data pair of predicted and measure BMD. The points close to the diagonal line suggests good calibration.

(a)

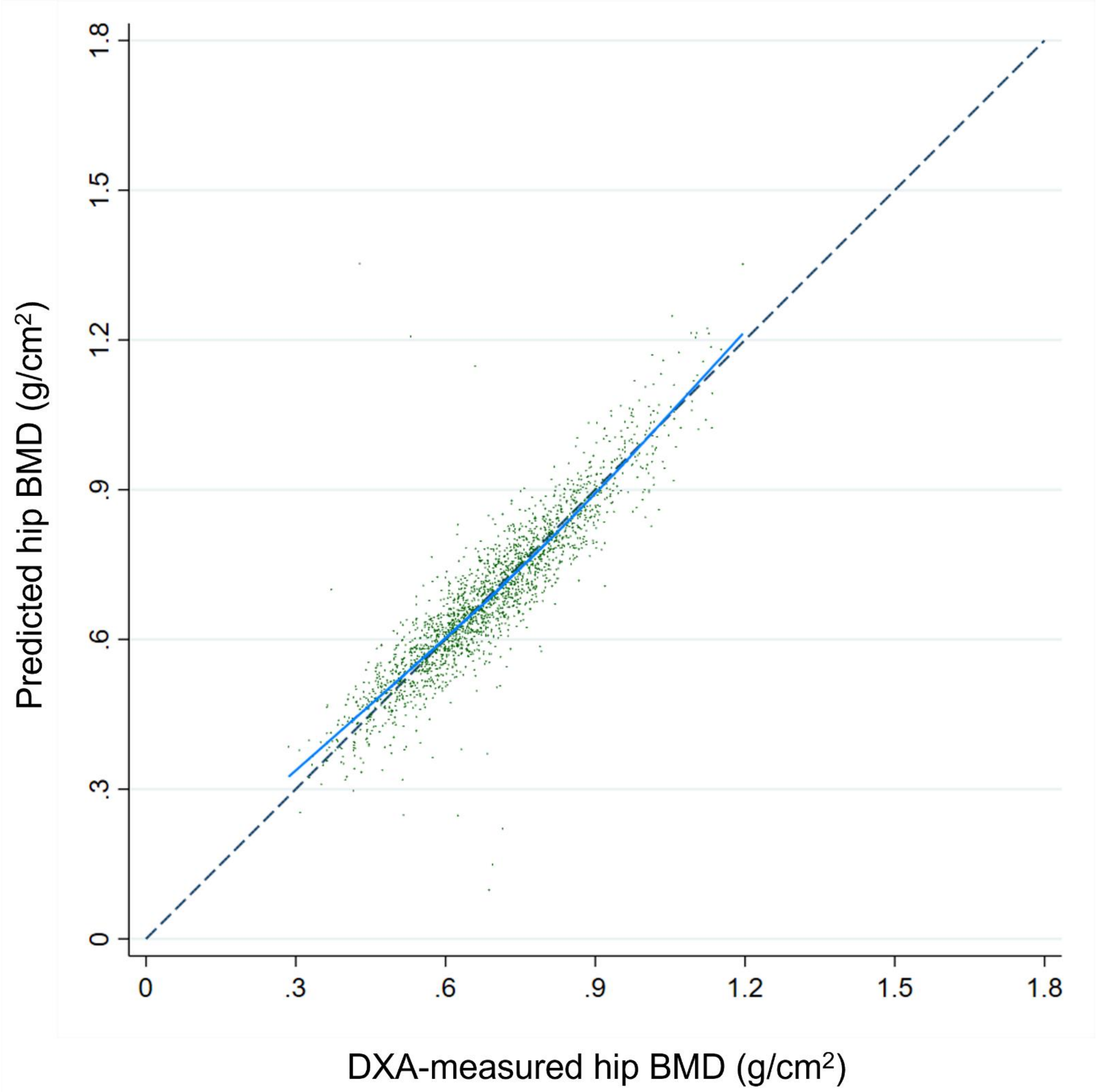

(b)

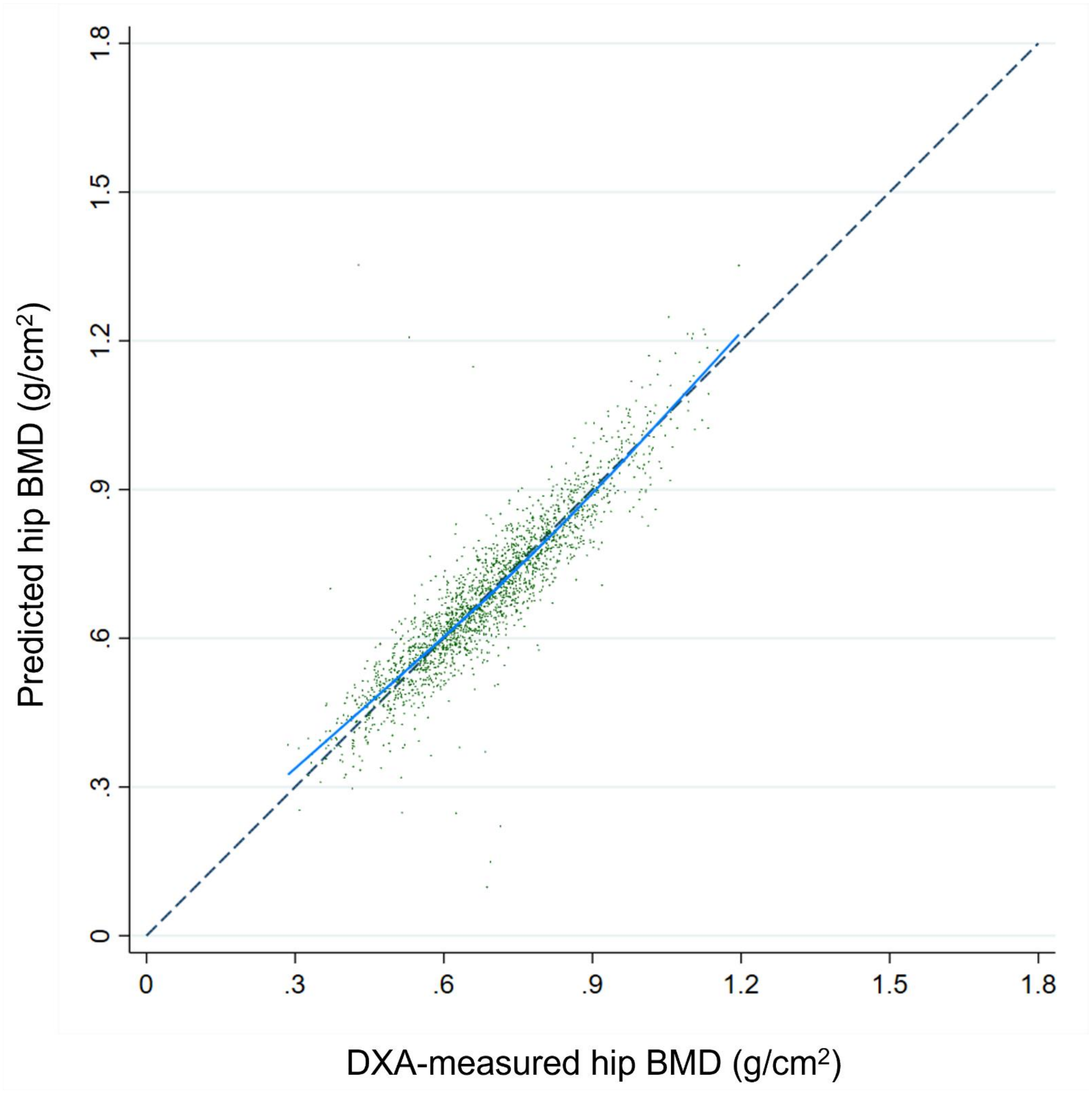
Predicted hip BMD (g/cm2)
DXA-measured hip BMD (g/cm2)
0
.3
.6
.9
1.2
1.5
1.8

Supplementary Figure 5 Data flow chart for the real-world clinical test. (a) hip BMD predictions and (b) spine BMD prediction. In this real-world test, we collected all patients receiving plain film for the hip and lateral plain film of the lumbar spine during January–May 2021. These images were fed into our tool and estimate the number (%) of images that can pass quality check, the distribution of predicted BMD, and the categorization based on the threshold reported in table 4.

(a)

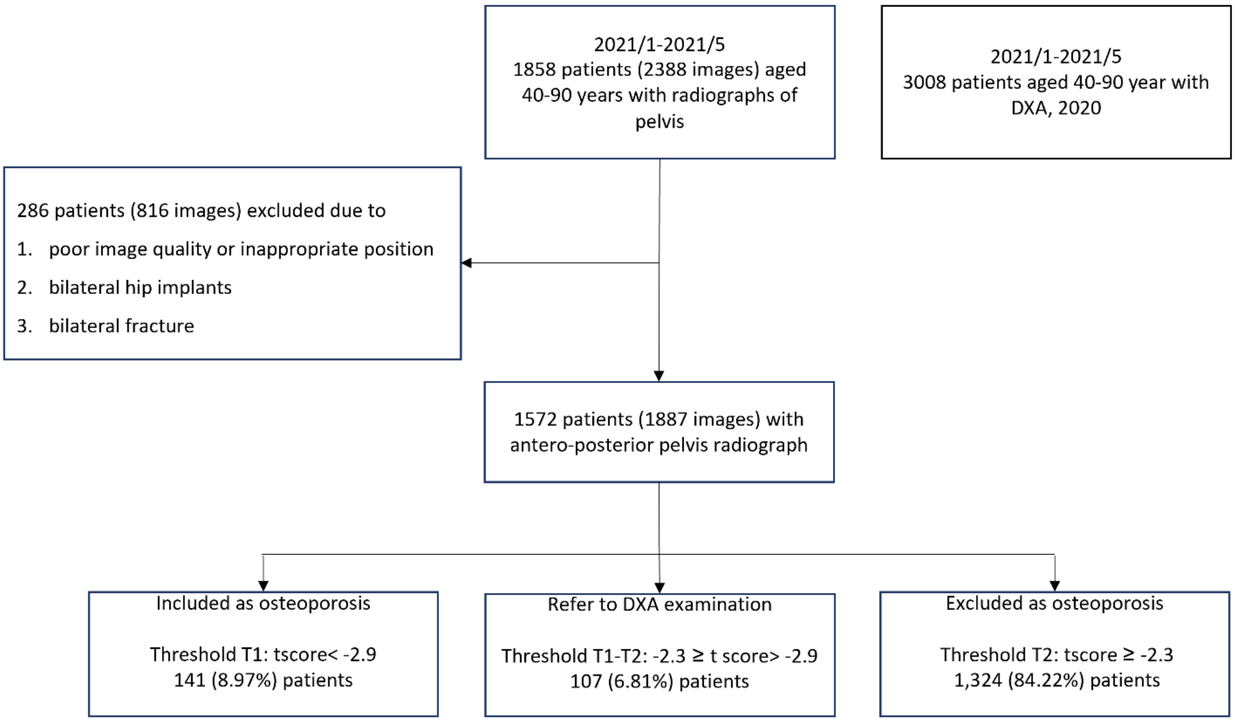

(b)

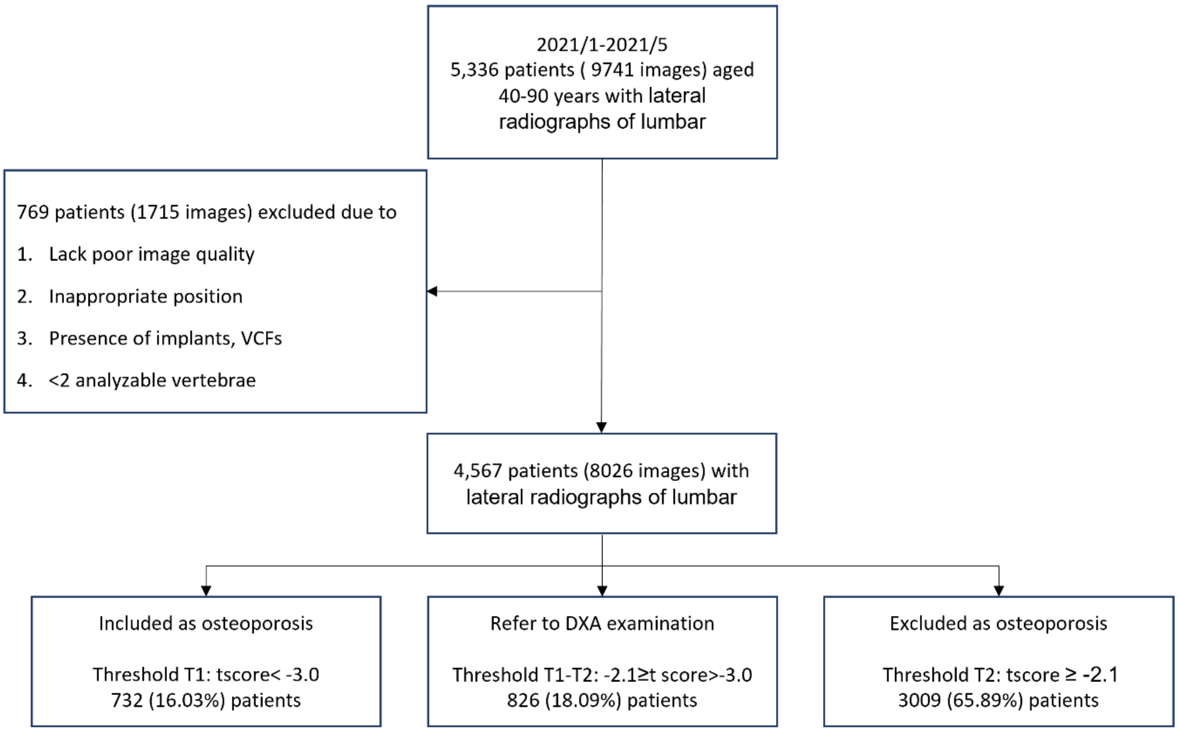
2021/1-2021/5
5,336 patients (9741 images) aged 40-90 years with lateral radiographs of lumbar
769 patients (1715 images) excluded due to
1. Lack poor image quality
2. Inappropriate position
3. Presence of implants, VCFs
4. <2 analyzable vertebrae
4,567 patients (8026 images) with lateral radiographs of lumbar
Included as osteoporosis
Threshold T1: tscore< -3.0
732 (16.03%) patients
Refer to DXA examination
Threshold T1-T2: -2.1≥t score>-3.0
826 (18.09%) patients
Excluded as osteoporosis
Threshold T2: tscore ≥ -2.1
3009 (65.89%) patients

Supplementary tables

Supplementary Table 1 Network performance across age and sex subsets

Discriminatory measures	Hip osteoporosis		10-year risk of major osteoporotic fracture >=20%		10-year risk of hip fracture >=3%		Lumbar vertebral osteoporosis	
Hologic								
	AUROC/AUPRC	OR (95% CI)	AUROC/AUPRC	OR (95% CI)	AUROC/AUPRC	OR (95% CI)	AUROC/AUPRC	OR (95% CI)
Female	0.96/0.90	64.84 (52.23–80.48)	0.97/0.84	82.44 (62.49–108.75)	0.97/0.97	82.79 (67.15–102.07)	0.91/0.91	35.23 (32.10–38.64)
Male	0.97/0.78	103.23 (55.98–109.35)	NA*	NA*	0.95/0.86	46.01 (31.13–68.00)	0.92/0.76	37.74 (30.55–46.63)
Age: <60 years	0.98/0.78	106.99 (46.98–243.69)	NA*	NA*	0.98/0.80	205.33 (74.08–569.14)	0.92/0.81	43.23 (35.17–53.15)
Age: 60-74 years	0.97/0.86	95.75 (63.59–144.19)	0.98/0.82	154.61 (87.01–274.73)	0.95/0.89	42.37 (31.43–57.11)	0.91/0.89	36.09 (31.92–40.82)
Age: 75-90 years	0.95/0.91	52.66 (41.18–67.34)	0.96 /0.84	69.22 (50.52–94.86)	0.97/0.97	59.93 (46.56–77.12)	0.91/0.92	31.93 (27.54–37.03)

* The number of patients with a 10-year risk of major osteoporotic fracture >=20% is less than 3.

Supplementary Table 2. Patient characteristics of the GE dataset for testing*

	Hip testing set	Spine testing set
Number	2060	3346
Female, n (%)	1554 (75.4)	2244 (67.1)
Mean age (sd), years	71.5 (12.1)	64.8 (11.7)
Median time (IQR) between DXA and radiographs	24 (6, 75)	28 (4, 95)
Mean BMI (sd), kg/m^2 *	23.7 (3.8)	24.5 (3.7)
Mean BMD (sd) g/cm^2 **	0.689 (0.160)	0.831 (0.183)**
Median T-score (IQR)	-1.5 (-2.3, -0.6)	-1.5 (-2.6, -0.3)**
Osteoporosis, n (%)	427(20.7)	975 (29.1)**

* Converted to the Hologic equivalent

** Calculated based on vertebrae with the lowest BMD.

Supplementary Table 3 BMD value conversion equations for GE Lunar to Hologic DXA scanners.

Measurement Site	Conversion Equation
Lumbar Spine	Hologic BMD= 0.918 x Lunar BMD – 0.038
Total Hip	Hologic BMD= 0.971 x Lunar BMD – 0.037

Supplementary Table 4. Summary of performance metrics of the predictive model for BMD* (GE DXA scanner)

Patient strata	Number of ROIs	Predicted vs. measured mean BMD (sd, g/cm^2); p	Correlation coefficient	Linear regression R^2, RMSE	Calibration slop, CITL	Bland-Altman bias (g/cm^2; sd)
The hip testing set (GE)*						
Overall	2060	0.690 (0.151) vs. 0.689 (0.160); p=477	0.90	0.81, 0.071	0.955, -0.001	-0.001 (0.071)
Female	1554	0.667 (0.143) vs. 0.670 (0.156); p<0.108	0.89	0.79, 0.071	0.968, 0.003	0.003 (0.068)
Male	506	0.762 (0.152) vs. 0.749 (0.161); p<0.001	0.91	0.82, 0.068	0.957, -0.013	-0.013 (0.069)
40-59 years	358	0.811 (0.158) vs 0.803 (0.135); p=0.076	0.85	0.72, 0.084	0.992, 0.008	0.008 (0.084)
60-74 years	685	0.726 (0.139) vs. 0.722 (0.147); p<0.03	0.91	0.82, 0.062	0.961, -0.005	-0.005 (0.062)
75-90 years	1017	0.626 (0.131) vs. 0.625 (0.137); p<0.001	0.86	0.74, 0.070	0.895, -0.002	-0.002 (0.072)
The spine testing set (GE)**						
Overall	11027	0.899 (0.172) vs. 1.036 (0.196); p<0.001	0.89	0.79, 0.090	1.011, 0.015	0.015 (0.090)
Female	7404	0.859 (0.1164) vs. 0.867 (0.187); p<0.001	0.88	0.788, 0.089	1.005, 0.008	0.008 (0.089)
Male	3623	0.978 (0.159) vs. 1.007 (0.179); p<0.001	0.86	0.73, 0.093	0.964, 0.028	0.028 (0.093)
40-59 years	3884	0.970 (0.155) vs. 0.991 (0.175); p<0.001	0.88	0.78, 0.083	0.993, 0.022	0.022 (0.083)
60-74 years	4598	0.878 (0.165) vs. 0.889 (0.188); p<0.001	0.88	0.77, 0.091	1.001, 0.011	0.011 (0.091)
75-90 years	2545	0.827 (0.168) vs. 0.838 (0.198); p<0.001	0.86	0.74, 0.100	1.015, 0.010	0.010 (0.100)
L1	2476	0.807 (0.153) vs. 0.821 (0.169); p<0.001	0.86	0.74, 0.085	0.946, 0.014	0.014 (0.085)
L2	3027	0.879 (0.164) vs. 0.887 (0.188); p<0.001	0.89	0.79, 0.087	1.020, 0.007	0.007 (0.087)
L3	2967	0.934 (0.163) vs. 0.959 (0.193); p<0.001	0.88	0.78, 0.091	1.046, 0.025	0.025 (0.091)
L4	2557	0.968 (0.165) vs. 0.980 (0.192); p <0.001	0.86	0.74, 0.097	1.005, 0.012	0.012 (0.097)

* GE BMD were converted to the Hologic equivalent.

** Calculated per eligible vertebrae.

Supplementary Table 5 Discriminatory performance (%) of the predicted BMD to classify hip/lumbar vertebral osteoporosis and high-risk groups for major osteoporotic or hip fractures (GE DXA scanner).

Discriminatory measures	Hip osteoporosis (T-score <= -2.5	Lumbar vertebral osteoporosis (vertebrae with the lowest T-score<= 2.5)	10-year risk of major osteoporotic fracture >=20%	10-year risk of hip fracture >=3%
Number of patients, %	427 20.7	975, 29.1	208, 10.4	922, 46.2
OR (95% CI)	66.38 (48.32–91.18)	39.47 (32.00–48.68)	101.10 (66.40–153.92)	102.20 (75.15–138.98)
AUROC/AUPRC	0.96/0.87	0.92/0.89	0.94/0.79	0.96/0.93
Accuracy (%; 95% CI)	91.4 (90.1–92.6)	87.6 (86.4–88.7)	94.8 (93.8–95.8)	91.0 (89.7–92.2)
Sensitivity (%; 95% CI)	78.9 (74.7–82.7)	80.6 (78.0–83.1)	74.0 (67.5–78.0)	89.9 (87.8–91.8)
Specificity (%; 95% CI)	94.7 (93.5–95.7)	90.5 (89.2–91.6)	97.3 (96.4–98.0)	92.0 (90.2–93.5)
PPV (%; 95% CI)	79.4 (75.8–82.7)	77.7 (75.4–79.8)	75.9 (70.2–80.7)	90.6 (88.7–92.2)
NPV (%; 95% CI)	93.5 (94.1–95.4)	87.6 (86.4–88.7)	97.0 (93.8–95.8)	91.4 (89.7–92.8)

Supplementary Table 6 Model performance in different x-ray machines and tanges of peak kilovolt (kV) for hip and lumbar radiographs.

Hip radiographs				Spine radiographs			
kVp distribution	n	%	Correlation coefficient	kVp distribution	n	%	Correlation coefficient
60-69 kV	1245	24.11	0.909	70-80 kV	5157	28.37	0.896
70-74 kV	995	19.27	0.921	90 kV	4637	25.51	0.889
75 kV	1394	26.99	0.917	95 kV	4747	26.12	0.898
Other	1530	29.63	0.922	Other	3634	19.99	0.889
Machine type	n	%	Correlation coefficient	Machine type	n	%	Correlation coefficient
Canon CDXI 710C	2576	49.88	0.919	Canon CDXI 710C	12337	67.88	0.896
Shimadzu MUX-100H	1161	22.48	0.914	Shimadzu MUX-100H	3501	19.26	0.885
Other	1427	27,63	0.917	Other	2337	12.86	0.887

Supplementary Table 7. Summary of performances using different backbone networks for hip BMD prediction (Hologic DXA).

Models	R-value	RMSE	R squared	Calib. slope	Calib. intercept	Mean diff	Std diff	Mean GT	Mean Pred	p-value
VGG-11	0.910	0.064	0.828	0.984	-0.002	0.002	0.064	0.689	0.690	0.034
VGG-16	0.906	0.065	0.821	0.966	0.013	-0.013	0.065	0.689	0.676	0.000
ResNet-18	0.917	0.062	0.839	0.985	-0.002	0.002	0.062	0.689	0.691	0.007
ResNet-34	0.917	0.062	0.841	0.984	-0.004	0.004	0.062	0.689	0.693	0.000
VGG-11 with age/gender	0.912	0.063	0.832	0.988	0.004	-0.004	0.063	0.689	0.684	0.000
VGG-16 with age/gender	0.909	0.064	0.827	0.973	-0.007	0.007	0.064	0.689	0.696	0.000
ResNet-34-retrain	0.917	0.062	0.841	0.982	-0.003	0.003	0.062	0.689	0.692	0.000

* Means were compared using student t test. Two-sided p values were reported.

Supplementary Table 8. Summary of performances using different backbone networks for spine BMD prediction (Hologic DXA).

Models	R-value	RMSE	Linear Regression R squared	Calibration slope	Calibration intercept (CITL)	Mean of difference	Std of difference	Mean GT BMD	Mean Pred BMD	p-value
VGG11	0.899	0.082	0.807	1.007	0.012	-0.012	0.082	0.839	0.827	0.000
VGG16	0.900	0.081	0.811	0.978	0.003	-0.012	0.082	0.839	0.837	0.000
ResNet18	0.893	0.084	0.798	0.971	-0.004	-0.003	0.081	0.839	0.843	0.000
ResNet34	0.896	0.083	0.803	0.980	0.000	0.003	0.084	0.839	0.839	0.168
VGG11 with age/gender	0.900	0.081	0.810	0.983	-0.001	0.001	0.081	0.839	0.840	0.002
VGG16 with age/gender	0.902	0.080	0.814	0.977	-0.007	0.006	0.081	0.839	0.846	0.000

* Means were compared using student t test. Two-sided p values were reported.

Supplementary Table 9. Pseudo code for hip BMD estimation in python style.

```
Input: hip X-ray image, I
```

```
def hip_bmd(I):
    """
    Estimate hip BMD from pelvic X-ray images
    """
    lmks = DagModel(I)            # Apply the DAG model
    score_fx = FrantureModel(I)    # Apply the fracture detection model
    score_implant = ImplantModel(I) # Apply the implant detection model

    box_l, box_r = roi_bounding_box(lmks) # Generate bounding boxes

    fx_l = roi_pooling(score_fx, box_l) > 0.5 # Fracture classification
    fx_r = roi_pooling(score_fx, box_r) > 0.5

    impl_l = roi_pooling(score_impl, box_l) > 0.5 # Implant classification
    impl_r = roi_pooling(score_impl, box_r) > 0.5

    if not fx_l and not impl_l:        # if no fracture and implant
        roi_l = roi_extraction(I, box_l) # extract ROI of the left hip
        bmd_l = BmdModel(roi_l)        # Apply the BMD model
    else:
        bmd_l = None

    in not fx_r and not impl_r:
        roi_r = roi_extraction(I, box_r)
        bmd_r = BmdModel(roi_r)
```

```
    else:
        bmd_r = None

    return bmd_l, bmd_r # return left and right hip BMDs
```

Supplementary Table 10. Pseudo code for spine BMD estimation in python style.

```
Input: Spine X-ray image, I
```

```
def spine_bmd(I):
    """
    Estimate spine BMD from lateral spine X-ray images
    """
    lmks = DagModel(I)            # Apply the DAG model
    score_fx = FractureModel(I)    # Apply the fracture detection model
    score_implant = ImplantModel(I) # Apply the implant detection model

    boxes = roi_bounding_box(lmks) # Generate bounding boxes as a dictionary

    bmd = {}
    for vert in ['L1', 'L2', 'L3', 'L4']:
        fx = roi_pooling(score_fx, boxes[vert]) > 0.5 # Fracture classification
        impl = roi_pooling(score_impl, boxes[vert]) > 0.5 # Implant classification
        ap_ratio, mid_ratio, height_ratio = \
            six_point_morph(boxes[vert]) # Calculate 6-point morphology metrics

        if not fx and not impl and \
            ap_ratio > 0.8 and mid_raio > 0.6 and height_ratio > 0.55: # If there is no fracture or implant and the morphology
is normal
            roi = roi_extraction(I, boxes[vert])
            bmd[vert] = BmdModel(roi)
        else:
            bmd[vert] = None
```

```
    return bmd['L1'], bmd['L2'], bmd['L3'], bmd['L4'] # Return 4 L-spine BMDs
```

Step 1: Open the project page https://gigantum.com/xraybmd/nc-bmd-cpu

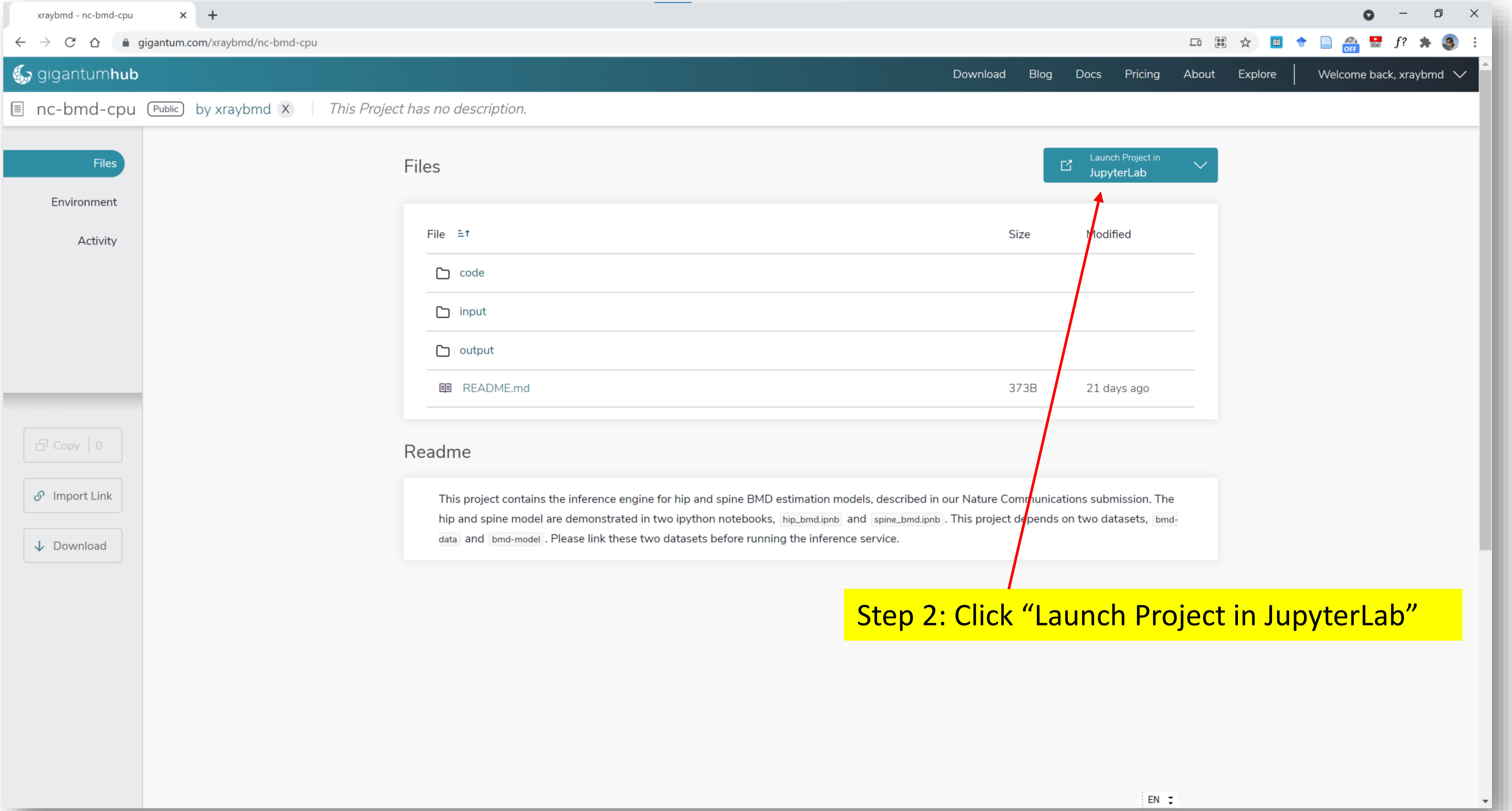

Step 2: Click "Launch Project in JupyterLab"

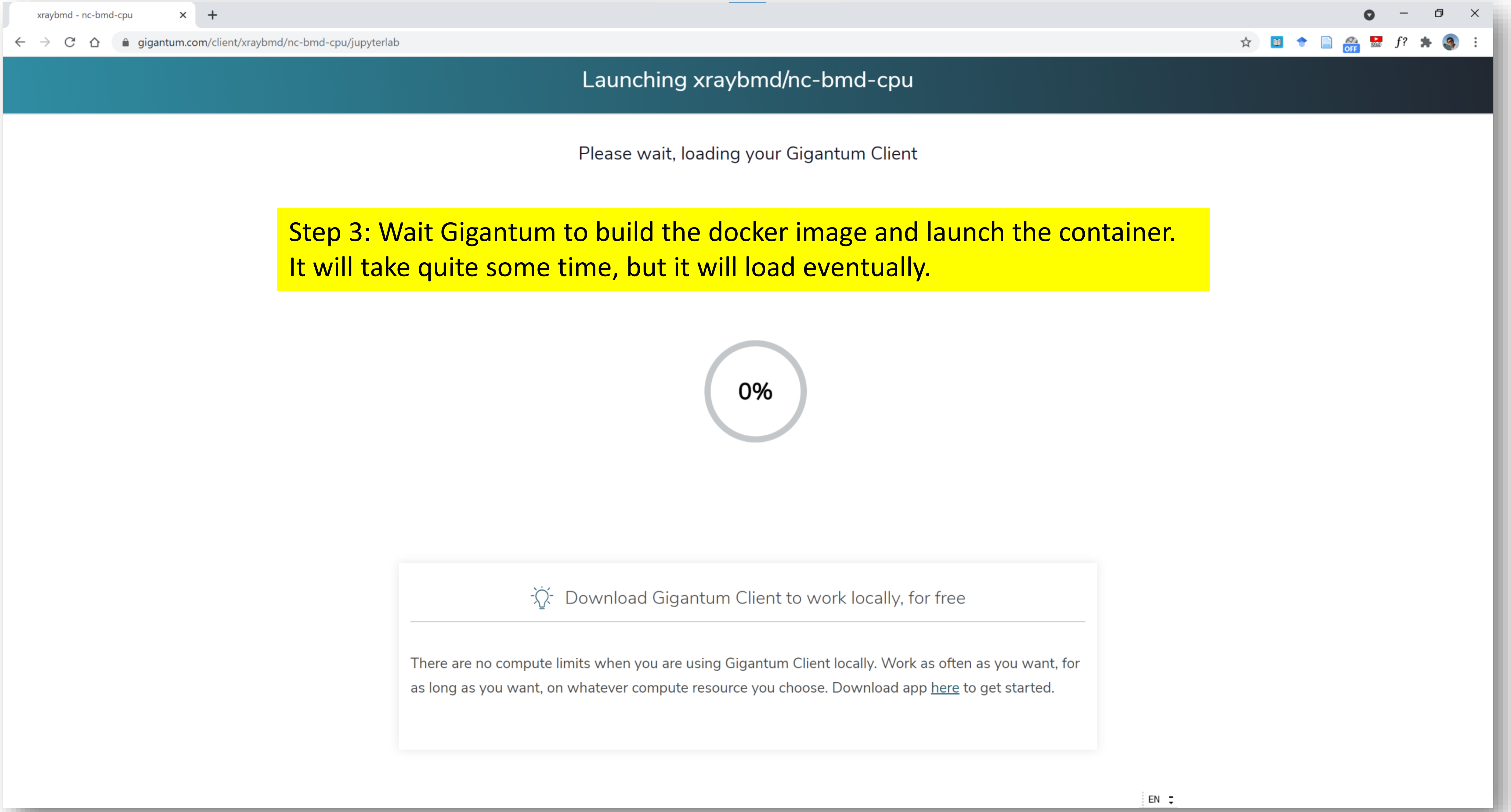
xraybmd - nc-bmd-cpu
gigantum.com/client/xraybmd/nc-bmd-cpu/jupyterlab
Launching xraybmd/nc-bmd-cpu
Please wait, loading your Gigantum Client
Step 3: Wait Gigantum to build the docker image and launch the container.
It will take quite some time, but it will load eventually.
0%
Download Gigantum Client to work locally, for free
There are no compute limits when you are using Gigantum Client locally. Work as often as you want, for as long as you want, on whatever compute resource you choose. Download app here to get started.
EN

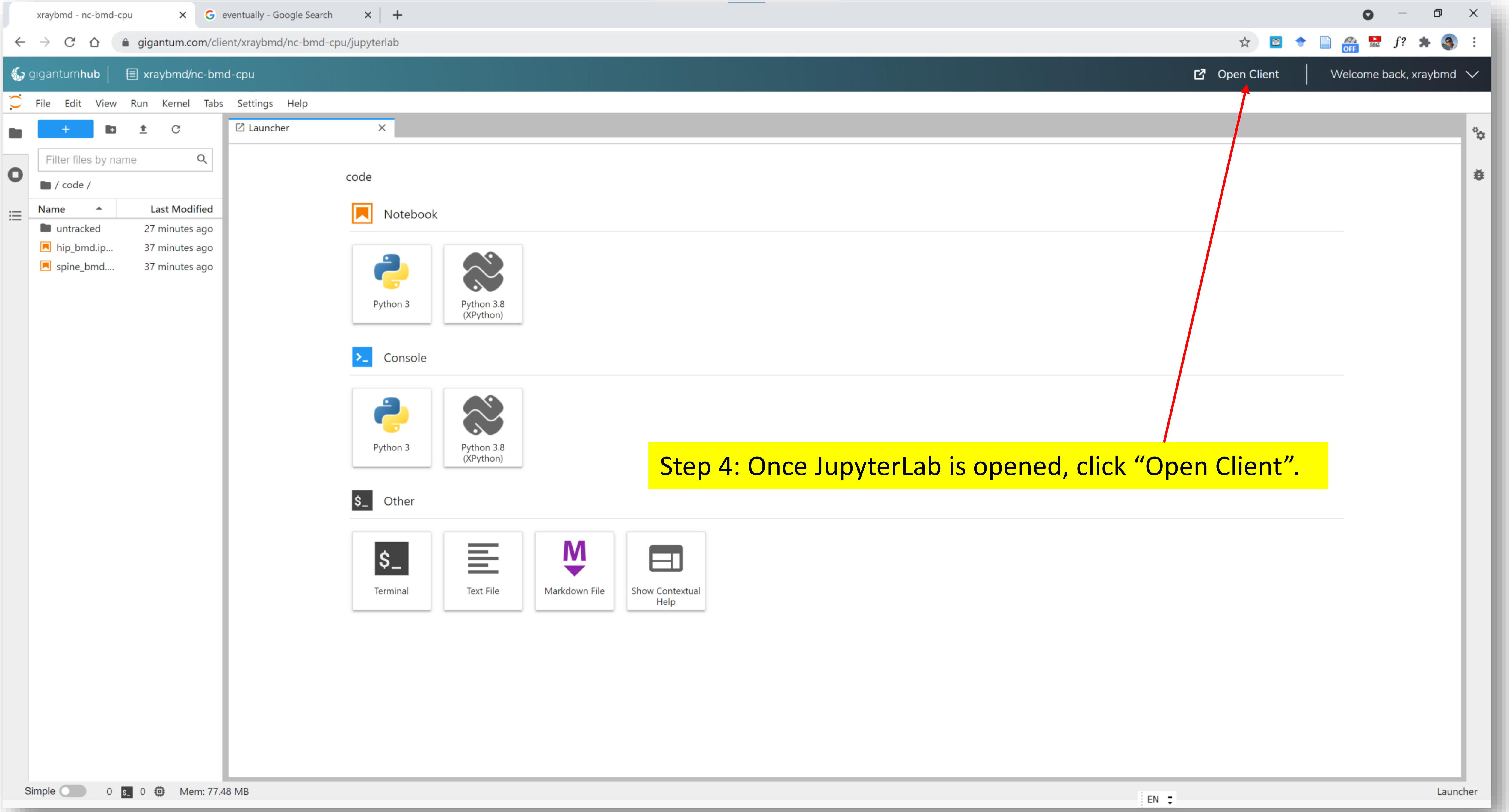

Step 4: Once JupyterLab is opened, click “Open Client”.

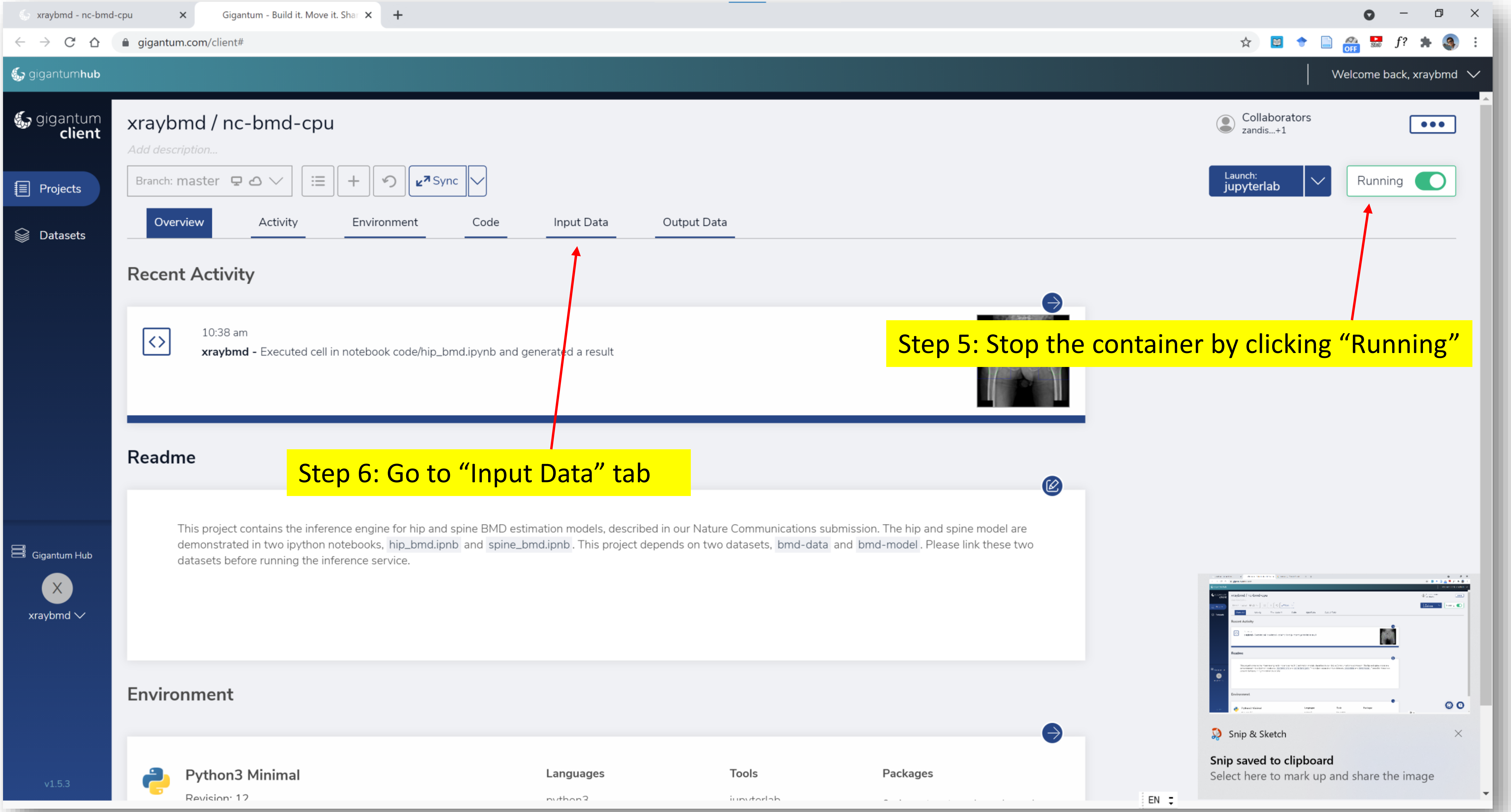
xraybmd - nc-bmd-cpu
Gigantum - Build it. Move it. Sha
gigantum.com/client#
gigantumhub
Welcome back, xraybmd
gigantum client
Projects
Datasets
xraybmd / nc-bmd-cpu
Add description...
Branch: master
Sync
Collaborators
zandis...+1
Launch: jupyterlab
Running
Overview
Activity
Environment
Code
Input Data
Output Data
Recent Activity
10:38 am
xraybmd - Executed cell in notebook code/hip_bmd.ipynb and generated a result
Step 5: Stop the container by clicking "Running"
Readme
Step 6: Go to "Input Data" tab
This project contains the inference engine for hip and spine BMD estimation models, described in our Nature Communications submission. The hip and spine model are demonstrated in two ipython notebooks, hip_bmd.ipnb and spine_bmd.ipnb. This project depends on two datasets, bmd-data and bmd-model. Please link these two datasets before running the inference service.
Gigantum Hub
xraybmd
v1.5.3
Environment
Python3 Minimal
Revision: 12
Languages
python3
Tools
jupyterlab
Packages
Snip & Sketch
Snip saved to clipboard
Select here to mark up and share the image
EN

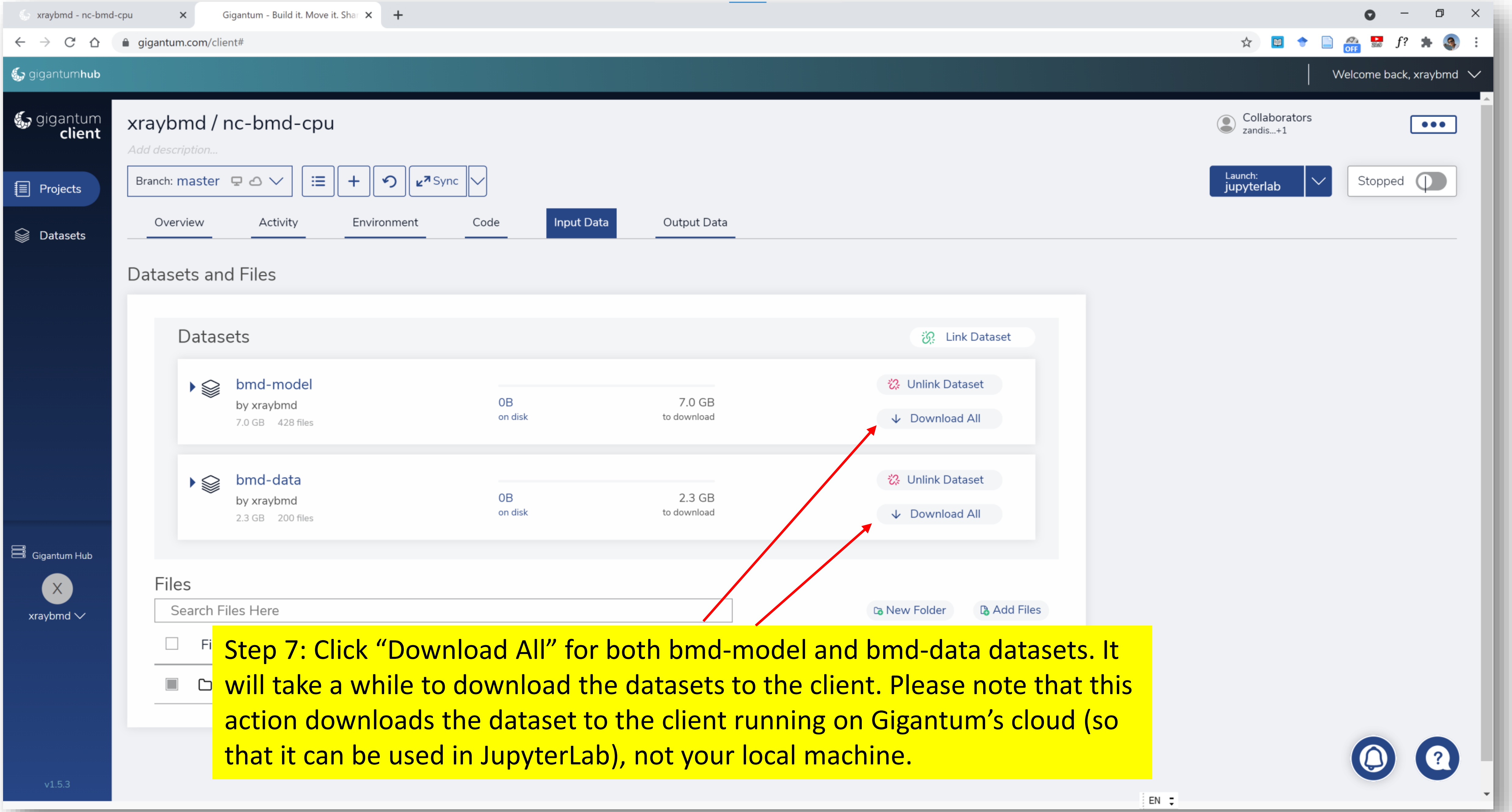

Step 7: Click “Download All” for both bmd-model and bmd-data datasets. It will take a while to download the datasets to the client. Please note that this action downloads the dataset to the client running on Gigantum’s cloud (so that it can be used in JupyterLab), not your local machine.

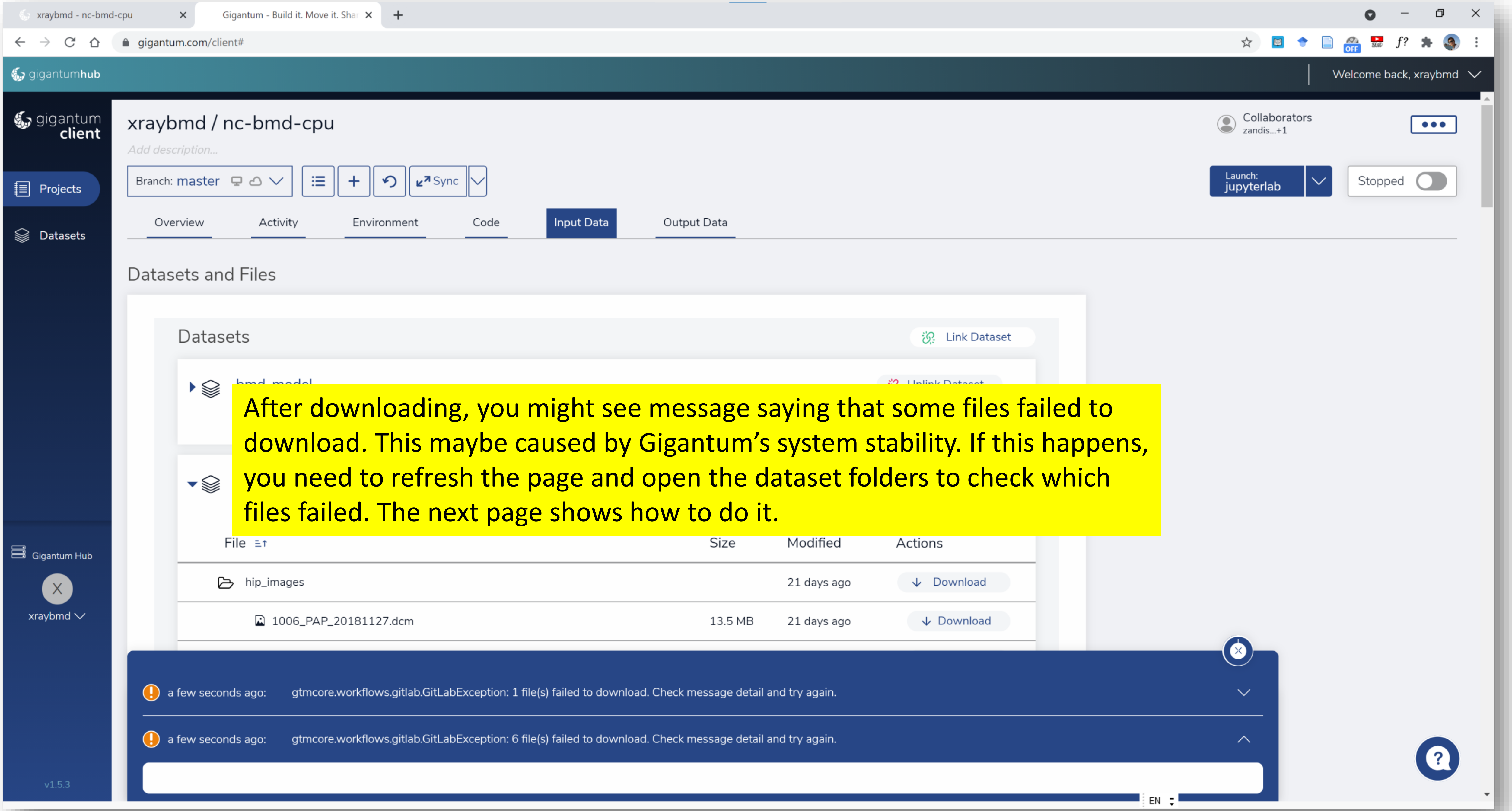

After downloading, you might see message saying that some files failed to download. This maybe caused by Gigantum's system stability. If this happens, you need to refresh the page and open the dataset folders to check which files failed. The next page shows how to do it.

Step 8: Download the files that failed to download by clicking the “Download” button. Files that are already downloaded shows “Downloaded”

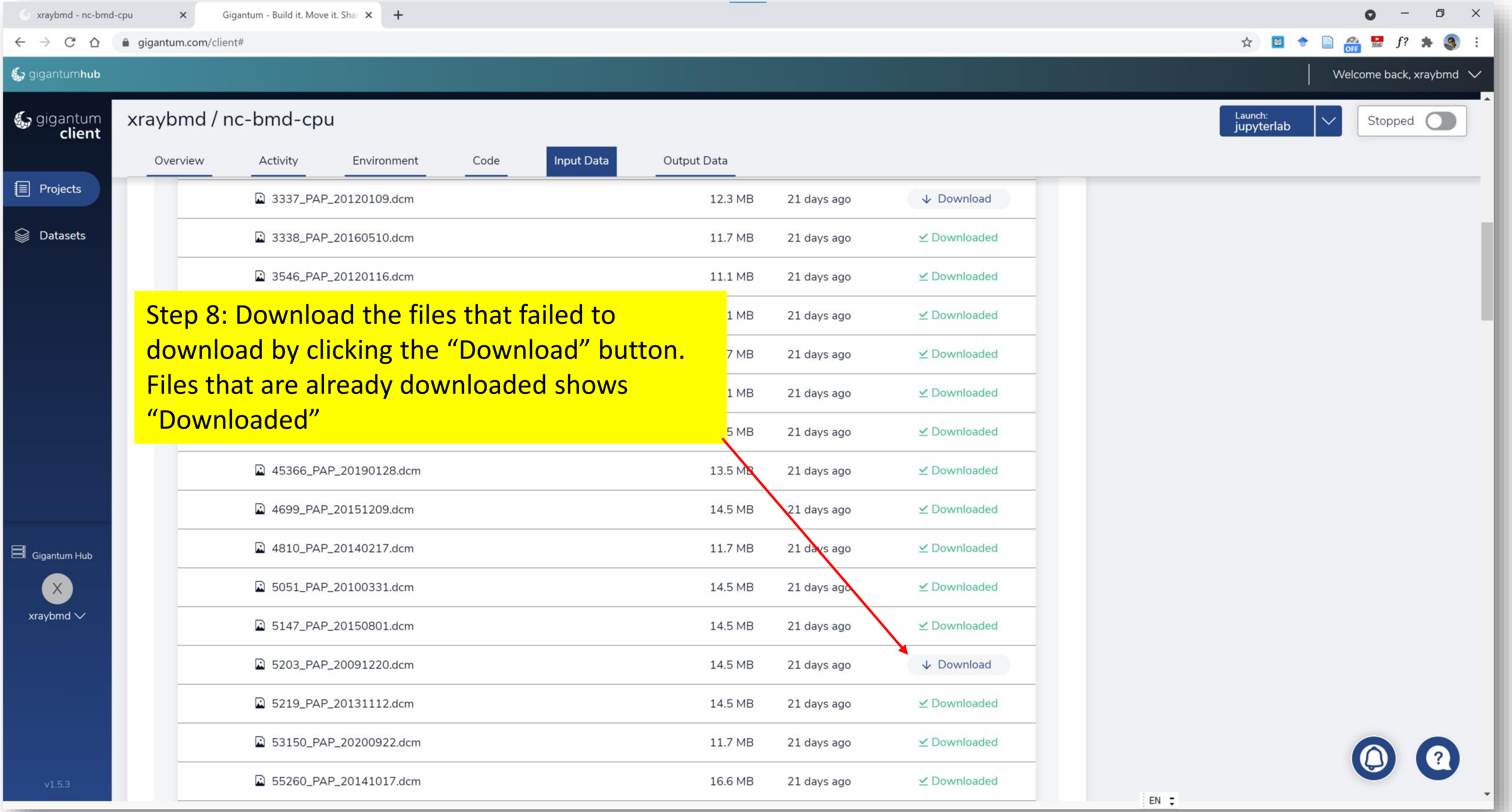

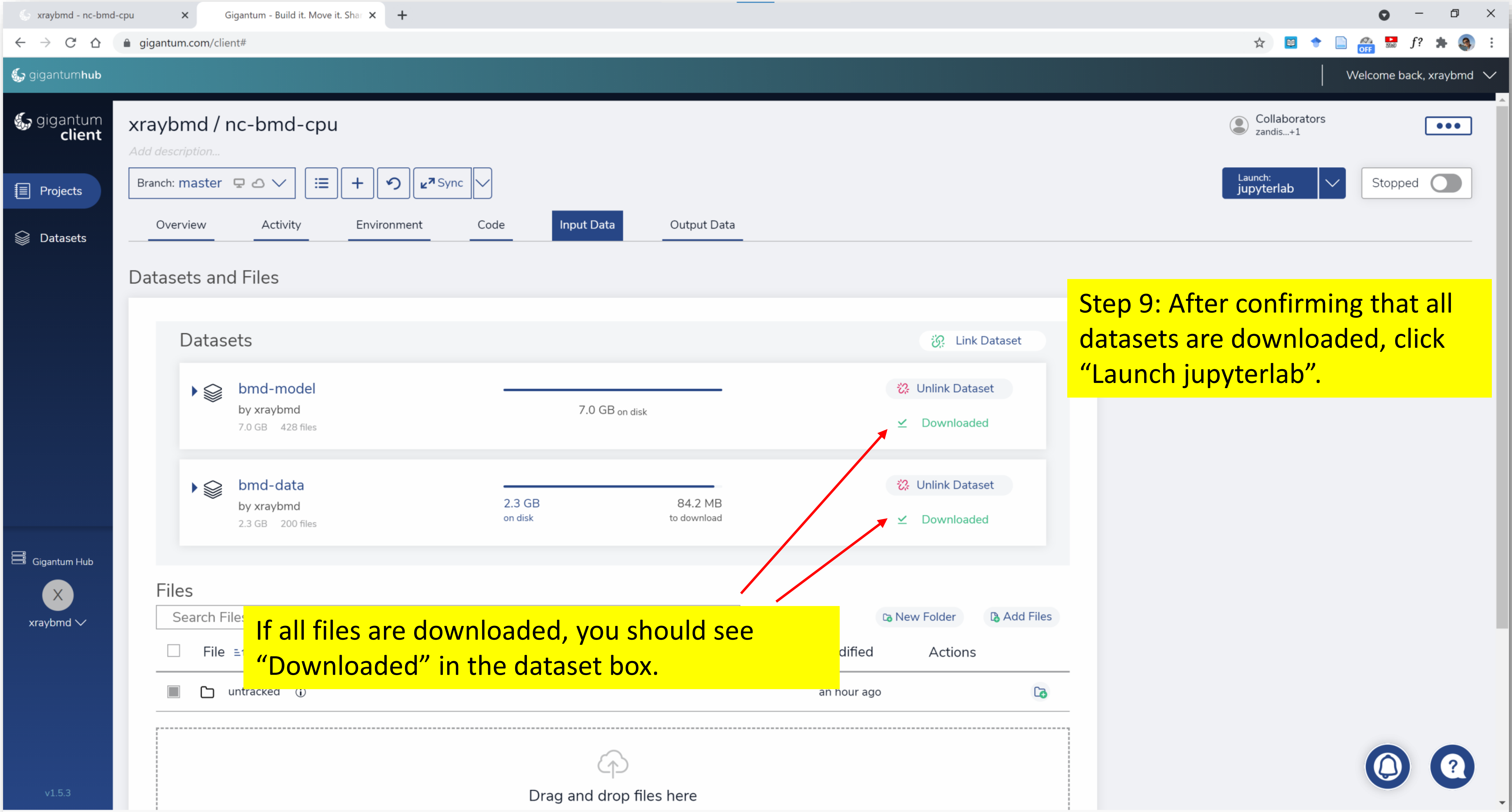

Step 9: After confirming that all datasets are downloaded, click "Launch jupyterlab".

If all files are downloaded, you should see "Downloaded" in the dataset box.

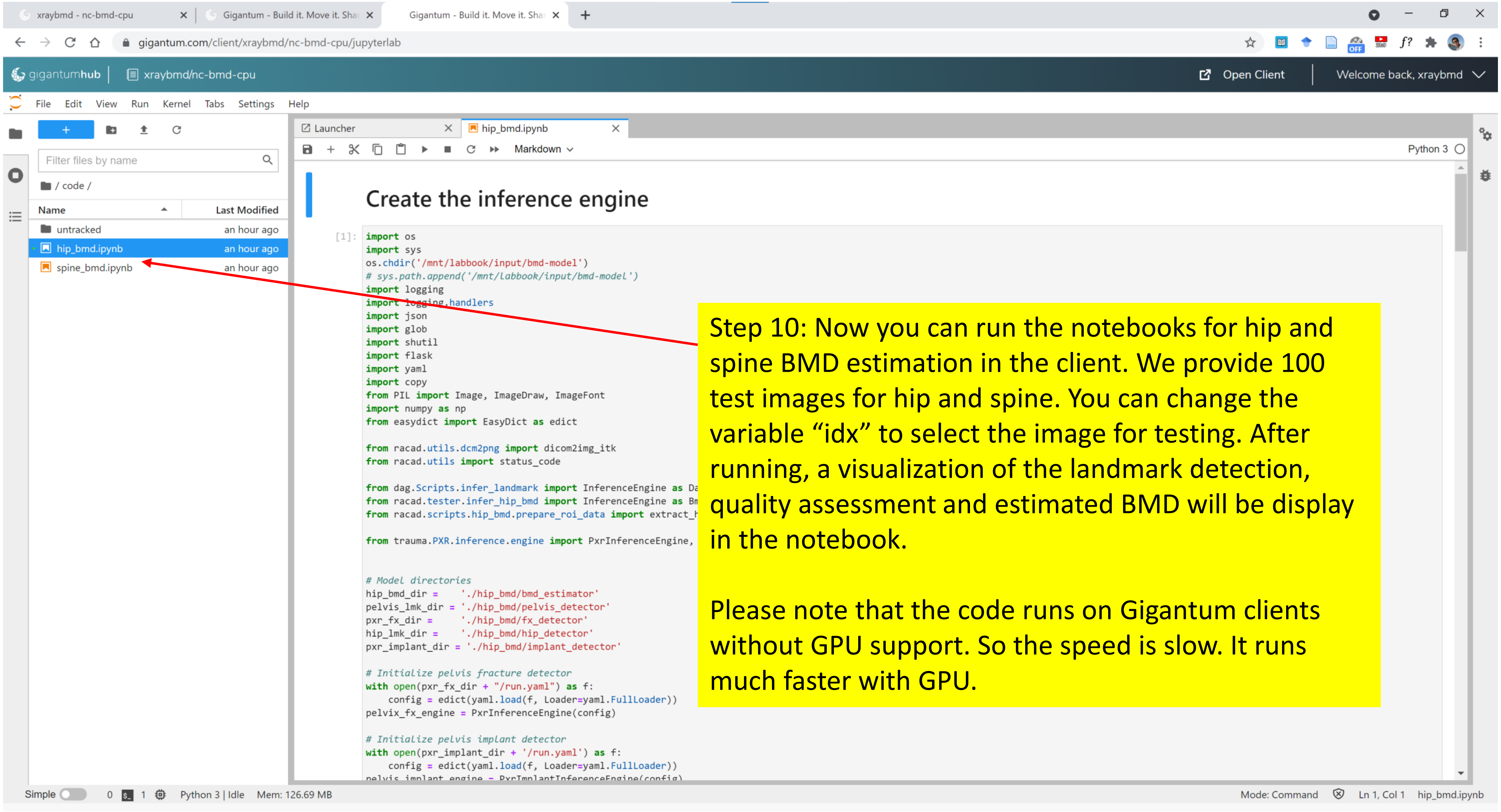

Step 10: Now you can run the notebooks for hip and spine BMD estimation in the client. We provide 100 test images for hip and spine. You can change the variable “idx” to select the image for testing. After running, a visualization of the landmark detection, quality assessment and estimated BMD will be display in the notebook.

Please note that the code runs on Gigantum clients without GPU support. So the speed is slow. It runs much faster with GPU.
