# Supplementary material for: Automated bone mineral density prediction and fracture risk assessment using plain radiographs via deep learning
Source: Nat Commun. 2021 Sep 16;12:5472. doi: 10.1038/s41467-021-25779-x (PMC8446034; doi:10.1038/s41467-021-25779-x)
Supplement: Supplementary file 2 — Reporting Summary [file 41467_2021_25779_MOESM2_ESM.pdf]

nature portfolio

Corresponding author(s): Kuo, Chang-Fu

Last updated by author(s): Aug 13, 2021

Reporting Summary

Nature Portfolio wishes to improve the reproducibility of the work that we publish. This form provides structure for consistency and transparency in reporting. For further information on Nature Portfolio policies, see our Editorial Policies and the Editorial Policy Checklist.

Statistics

For all statistical analyses, confirm that the following items are present in the figure legend, table legend, main text, or Methods section.

n/a	Confirmed	
☐	☒	The exact sample size (n) for each experimental group/condition, given as a discrete number and unit of measurement
☐	☒	A statement on whether measurements were taken from distinct samples or whether the same sample was measured repeatedly
☐	☒	The statistical test(s) used AND whether they are one- or two-sided<br>*Only common tests should be described solely by name; describe more complex techniques in the Methods section.*
☐	☒	A description of all covariates tested
☐	☒	A description of any assumptions or corrections, such as tests of normality and adjustment for multiple comparisons
☐	☒	A full description of the statistical parameters including central tendency (e.g. means) or other basic estimates (e.g. regression coefficient) AND variation (e.g. standard deviation) or associated estimates of uncertainty (e.g. confidence intervals)
☐	☒	For null hypothesis testing, the test statistic (e.g. F, t, r) with confidence intervals, effect sizes, degrees of freedom and P value noted<br>*Give P values as exact values whenever suitable.*
☒	☐	For Bayesian analysis, information on the choice of priors and Markov chain Monte Carlo settings
☒	☐	For hierarchical and complex designs, identification of the appropriate level for tests and full reporting of outcomes
☐	☒	Estimates of effect sizes (e.g. Cohen's d, Pearson's r), indicating how they were calculated

Our web collection on statistics for biologists contains articles on many of the points above.

Software and code

Policy information about availability of computer code

Data collection	python 3.6, Pytorch v1.3, FRAX Desktop Multi-Patient Entry, version 4.0
Data analysis	Stata 16.1

For manuscripts utilizing custom algorithms or software that are central to the research but not yet described in published literature, software must be made available to editors and reviewers. We strongly encourage code deposition in a community repository (e.g. GitHub). See the Nature Portfolio guidelines for submitting code & software for further information.

Data

Policy information about availability of data

All manuscripts must include a data availability statement. This statement should provide the following information, where applicable:

- Accession codes, unique identifiers, or web links for publicly available datasets
- A description of any restrictions on data availability
- For clinical datasets or third party data, please ensure that the statement adheres to our policy

The sample testing imaging data generated in this study have been deposited in the Zenodo database (doi: 10.5281/zenodo.5216219). The full original imaging data are available under restricted access for the policy of the Chang Gung Memorial Hospital and data privacy laws, access can be obtained by a reasonable request to the corresponding author. Use of data is limited to research purpose and redistribution of data is not allowed.

Field-specific reporting

Please select the one below that is the best fit for your research. If you are not sure, read the appropriate sections before making your selection.

☒ Life sciences ☐ Behavioural & social sciences ☐ Ecological, evolutionary & environmental sciences

For a reference copy of the document with all sections, see nature.com/documents/nr-reporting-summary-flat.pdf

Life sciences study design

All studies must disclose on these points even when the disclosure is negative.

Sample size	We collected all patients (184,339) patients with at least one central DXA from January 2006 to December 2020 in CGMH and were aged 40–90 years on the DXA index date. The study population was also required to have adequate radiographs of the pelvis or lumbar spine within 180 days from the index date. These DXA-radiograph pairs were the primary data source for the study. We did not calculate sample size, but based on knowledge of the prior similar studies, we have the largest study population to date. Therefore, the sample size is sufficient to achieve the conclusion.
Data exclusions	Some medical conditions may affect the hip and vertebra anatomy, making plain films unsuitable for BMD estimation. The most common conditions include implantation and fracture. Therefore, we conducted an automated quality assessment to exclude hips and vertebrae with implants or fractures unsuitable for BMD prediction. For the hip, We detect hip fracture and implant (joint prosthesis, screws, plates, or cement) in the quality assessment process and exclude them from the downstream BMD estimation. The adult official positions of the ISCD advise excluding vertebrae that are abnormal and non-assessable or have a more than a 1.0 T-score difference between the vertebra in question and adjacent vertebrae. Therefore, the automated quality assessment procedure for spine radiographs is performed in three steps: implant and VCF detection, six-point morphology analysis and assessment for T-score of nearby vertebrae. These exclusion criteria were pre-established.
Replication	We tested our primary findings using the GE DXA data and data from external source. The performance remains robust. The results are reported in the supplementary material.
Randomization	After the exclusion of inadequate plain films, model building and testing were performed based on a cohort of 10797 patients with at least one Hologic DXA-pelvis radiograph pair and 25482 patients with at least one lateral radiograph of the lumbar spine–DXA pair (Figure S1). These patients were randomly split into the testing and training set by Simple Random Sampling. In simple random sampling, each unit has an equal probability of selection, and sampling is without replacement. Without-replacement sampling means that a unit cannot be selected more than once.
Blinding	This study is not an interventional study. The randomization process in our study aims to do training/testing data split. The training set was used to build up a model which is to test on the testing set. In addition, the ground truth is based on the DXA measurement, the predicted values is produced by the model. In the process, the investigator has no influence on the assessment of BMD. Blinding procedure is not possible in our study and will not prevent the occurrence of bias.

Reporting for specific materials, systems and methods

We require information from authors about some types of materials, experimental systems and methods used in many studies. Here, indicate whether each material, system or method listed is relevant to your study. If you are not sure if a list item applies to your research, read the appropriate section before selecting a response.

Materials & experimental systems

n/a	Involved in the study	
☒	☐	Antibodies
☒	☐	Eukaryotic cell lines
☒	☐	Palaeontology and archaeology
☒	☐	Animals and other organisms
☐	☒	Human research participants
☐	☒	Clinical data
☒	☐	Dual use research of concern

Methods

n/a	Involved in the study	
☒	☐	ChIP-seq
☒	☐	Flow cytometry
☒	☐	MRI-based neuroimaging

Human research participants

Policy information about studies involving human research participants

Population characteristics	The final study population included 5164 patients (3997 women [77.4%], mean age, 72.2 [standard deviation, SD, 11.2] years) in the hip testing set and 18175 patients (14469 women [79.6%], mean age, 67.1 [SD, 10.6] years) in the spine testing set.
Recruitment	We retrospectively identified patients with both DXA and hip/spine radiographs with an interval of 180 days. From 2006 to 2020, 30958 and 86977 patients aged 40-90 years with paired DXA-pelvis or paired DXA-lateral radiographs of the lumbar spine (18.6% and 18.2% of patients with hip or lumbar spine radiographs) were screened to identify hip and spine cohorts for analysis. Of these, 18097 and 58149 patients in the respective cohorts were excluded due to a DXA-radiograph interval >180 days, lack of detailed reports, inadequate image quality, positions, or analyzable ROIs. The final cohorts included 10797 patients with Hologic DXA-hip radiograph pairs and 25482 patients with Hologic DXA- spine radiograph pairs.
Ethics oversight	Institutional Review Board of Chang Gung Memorial Hospital

Note that full information on the approval of the study protocol must also be provided in the manuscript.

Clinical data

Policy information about clinical studies

All manuscripts should comply with the ICMJEguidelines for publication of clinical research and a completedCONSORT checklist must be included with all submissions.

Clinical trial registration	This study is not an interventional clinical trial.
Study protocol	The protocol of this study is well explained in the main manuscript. As mentioned previously, we conducted a retrospective data collection, split it into training and testing sets. The models based on the training set are testing in the testing set and external sets. We conducted an observation study to assess the impact of our automated tool on the osteoporosis screening.
Data collection	This study was performed using data from CGMH, the largest private hospital system in Taiwan, which includes seven acute hospitals with 10050 beds that received 8.2 million outpatient visits and 2.4 million inpatient care visits. The study population consisted of 184,339 patients with at least one central DXA from January 2006 to December 2020 and were aged 40–90 years on the DXA index date. The study population was also required to have adequate radiographs of the pelvis or lumbar spine within 180 days from the index date. For patients with multiple DXA and plain film radiographs, the earliest pair was used. We performed a quality check for plain films to ensure that these images were suitable for BMD prediction; after the exclusion of inadequate plain films, model building and testing were performed based on a cohort of 10797 patients with at least one Hologic DXA-pelvis radiograph pair and 25482 patients with at least one lateral radiograph of the lumbar spine–DXA pair (Figure S1). Patients with GE DXA-plain film pairs were used as the separate testing sets (hip testing set, n = 2060; spine testing set, n = 3346). We also include 34 pairs of GE DXA-hip radiographs and 179 pairs of DXA-lumbar spine radiographs from the Wuhan Hospital of Traditional Chinese Medicine to do external validation.<br>We also tested the algorithms in a clinical setting to ascertain the number and proportions of patients with hip or spine radiographs who may benefit from the tool. The algorithms were packaged in docker containers and implemented on the PACS-linked inference platform of CGMH, based on the Nvidia Triton architecture. We tested the model using consecutive radiographs conducted between January 2021 and May 2021.
Outcomes	Evaluation of all performance measures was performed only on the test datasets. The Bland–Altman plot visualized the agreement between predicted and measured BMD scores, and Pearson's correlation coefficient was calculated. The tool's calibration was evaluated by comparing the mean risk calculated based on predicted BMD and the mean risk based on DXA-measured BMD. The following measures were calculated to evaluate the overall calibration: calibration slope and calibration-in-the-large. Osteoporosis results were considered positive when T-score ≤ −2.5. Ten-year probabilities of major fracture and hip fracture with total hip BMD were calculated for each patient using the FRAX tool with risk estimators specific to the Taiwanese population (https://www.sheffield.ac.uk/FRAX/; FRAX Desktop Multi-Patient Entry, version 4.0). The FRAX parameters used in this study include age, sex, weight, height, and BMD. FRAX risks with and without BMD were calculated separately. For each patient, the lowest BMD was used to calculate the T-score and FRAX risk. Ten-year risk scores of ≥ 3% for hip fracture and ≥ 20% for major osteoporotic fracture were considered high-risk, based on the intervention threshold established in the Taiwan Osteoporosis Practice Guidelines and the recommendations of the National Osteoporosis Foundation. The overall discriminative abilities to discern osteoporosis and high-risk patients were evaluated using the area under the reciver-operator curve (AUROC) and area under the precision-recall curve (AUPRC). Other measures were also calculated, including sensitivity, specificity, positive predictive value, and negative predictive value.
